# Measuring Dental Caries in the Mixed Dentition by ICDAS

**DOI:** 10.1155/2011/150424

**Published:** 2011-10-31

**Authors:** Eino Honkala, Riina Runnel, Sisko Honkala, Jana Olak, Tero Vahlberg, Mare Saag, Kauko K. Mäkinen

**Affiliations:** ^1^Faculty of Dentistry, Kuwait University, P.O. Box 24923, Safat 13110, Kuwait; ^2^Institute of Stomatology, University of Tartu, Raekoja Plats 6, 51003 Tartu, Estonia; ^3^Faculty of Medicine, University of Turku, 20014 Turku, Finland

## Abstract

Caries has traditionally been assessed with WHO criteria including only obvious caries lesions. ICDAS has been developed to detect also the enamel caries lesions. This study aims to study caries and the associations of the number of caries lesions between the permanent and primary molars with ICDAS in the mixed dentition of the first and second grade primary school children. The clinical examinations of 485 children were conducted by four examiners with high reproducibility (inter- and intraexaminer kappas >0.9). The mean number of caries lesions—especially dentine caries—seemed to be higher in the second primary molars than in the first permanent molars. There were significant correlations between the number of lesions on occlusal and lingual surfaces between the primary and permanent molars. Enamel caries lesions, restorations, and caries experience did not increase according to age. Therefore, caries might be increasing in this population. As a conclusion, ICDAS recording seems to give appropriate information from the occurrence of caries lesions and its correlations between the primary and permanent teeth and surfaces.

## 1. Introduction

Reliable, reproducible, and practical detection and assessment of dental caries lesions as an outcome of dental caries disease has been a challenge for a long time [1, 2]. The lesions can be detected on all surfaces of the primary, permanent, and mixed dentitions. Surface lesions can then be counted according to the type of the teeth (incisors, canines, premolars, and molars) or according to the surfaces (occlusal, proximal, and free smooth surfaces). Mixed dentition stage normally includes the age groups from 6 to 12 years, when the permanent teeth are erupting and the primary teeth exfoliating. The exfoliation is a special problem in the prospective clinical caries trials, when the tooth and surfaces need to be present in both of the examinations. However, also cross-sectional clinical studies can still give important descriptive information for monitoring the trends and for giving dental health a visibility for policy makers [3]. The mixed dentition is the first stage to study an association of the number of caries lesions between the primary and permanent teeth. Several studies have shown very clear correlations in caries experience between the primary and permanent teeth [4–9]. 

Traditionally, caries has been measured by DMFT/S index, where only teeth or surfaces with cavitated lesions extending into the dentine have been counted [10, 11]. Over the years, DMFT index has been criticized for several reasons [12]:

1. diagnosis of caries lesions has been shown to be unreliable,
2. the reason for extraction for caries is very difficult to confirm at the point of examination,
3. secondary caries lesions on surfaces with restorations are not counted,
4. the activity of the lesions is not determined,
5. enamel caries lesions are not included,
6. DMF values are not related to the number of teeth/surfaces at risk,
7. DMF index gives an equal weight to missing teeth, untreated caries, or restored teeth,
8. DMF index can overestimate caries experience by teeth with PRR (preventive resin restorations) or with cosmetic restorations,
9. DMFT index is of a little use for estimating treatment needs,
10. DMF index does not include sealants.

One additional problem with DMF index has been the skewed distribution of caries experience, which could be measured by using the significant caries index (SiC) [13]. SiC measures the mean DMF score among the third of the subjects, who are the most affected by caries. The International Caries Detection and Assessment System (ICDAS) was developed to include early enamel caries lesions according to the stage of their progression as well as to categorize the “obvious,” dentine caries lesions according to their progression [2, 12, 14, 15]. The validity and reproducibility of ICDAS has already been tested in several in vitro [16–18] and clinical studies [19–21]. There are also some large epidemiological studies conducted using ICDAS [22, 23]. ICDAS is now the international recommendation for dental health surveys [24]. There are still only a few studies, where ICDAS has been used in the prospective study design [25]. This study was concluded as a baseline for the clinical trial in Southeast Estonia. 

This baseline study aims to find out the distributions of the caries lesions and their associations between the first permanent molars and the second primary molars with the ICDAS criteria among the first and the second grade schoolchildren with mixed dentition.

## 2. Subjects and Methods

This study was conducted at the University of Tartu Dental Clinic in January 2008. The samples of the first and second grade pupils in Southeast Estonia were included in this study. The schools were selected from all primary schools of this region. The sample (*N* = 522) was drawn according to the geographical area (county) and the size of the school (small, average, and large). The written informed consent forms were signed by the parent/caretaker. Altogether 485 children (93%) participated in the clinical examinations, 45.6% being boys and 54.4% girls. Very few parents/caretakers did not want their child to participate, but some children were absent on the day of the clinical examination. The mean age was 7.8 years (SD = 0.35) in the first grade and 8.8 years (0.38) in the second grade (Table 1). 

All the examinations were conducted in standard dental chairs of the Department of Stomatology of the University of Tartu. The clinical examinations were conducted by four trained and calibrated examiners. Before the study, a 90-minute e-learning program of ICDAS system was sent to all examiners. The examiners were senior academic staff with a long clinical experience. After the e-learning training, calibration sessions were arranged in the examination site during two days before starting the study. After a joint discussion about the ICDAS criteria, four children were examined by all four examiners and the caries diagnoses compared with each examiner and finally verified clinically by reexamining each child by all examiners together. Another four children were examined by the similar way. On the second day, altogether 25 children outside the sample were studied twice; by one examiner (S. Honkala, R. Runnel, J. Oulak) and by the examiner 1 (E. Honkala) and the differences were discussed. During the study, the recorders scheduled 10 children to be examined twice by each examiner and another 10 children by each examiner and by the examiner 1. All the reexaminations were done at the same day as the first examinations, because all the children came to Tartu by a bus from their school. The inter- and intraexaminer repeatability was high; all the weighted kappa values being >0.9, as reported earlier [26]. 

The children were allocated randomly to the examiners. The children were given a toothbrush and toothpaste and requested to brush their teeth before the examination. The clean teeth were then assessed according to the ICDAS criteria [14], first as wet and then after drying with compressed air. Dental mirror and WHO periodontal probe were used as visual-tactile aids in assessing the surfaces. Two digit ICDAS codes were determined for each tooth surface of the mixed dentition. 

The data were installed with Excel software and analyzed by SPSS (version 17.0) and SAS (version 9.2) programs. The first digit of the ICDAS code describes the treatment provided, and the second digit is the actual caries code. The occlusal surfaces with full or partial sealants were considered as healthy (ICDAS caries code 0). In the analyses of the caries indices ICDAS caries codes 1, 2, and 3 were counted together as a measure of enamel caries (D_1–3_) and 4, 5, and 6 as dentine caries (D_4–6_). Caries experience (D_4–6_MFT and D_4–6_MFS) was calculated as a total number of teeth/surfaces with dentine caries, or/and treated caries (FT/FS) and missing teeth/surfaces (MT/MS) because of caries. The children with D_4–6_MFS = 0 were determined as free from obvious decay. 

The distributions of the ICDAS codes according to the tooth surfaces were presented by counting the means of the prevalence on the right and the left side of the mouth together. The children, who had their permanent second premolars erupted (6-7%) were excluded, when calculating the distribution of the different ICDAS codes of the primary teeth. The association between the first permanent molars and the second primary molars was analyzed by Spearman correlation coefficient (*r*). The number of missing surfaces (extracted due to caries, missing for other reason, or unerupted) was very small, 1–4%. The mean caries indices were analyzed according to the age and grade by Kruskal-Wallis test and according to school by Mann-Whitney *U* test. 

## 3. Results

There was a clear tendency that the lower ICDAS caries codes (1–3) were more prevalent in the permanent molars than in the primary molars and the higher ICDAS codes (4–6) more prevalent in the second primary molars than in the permanent molars (Table 2). The most prevalent caries code (>0) was code 2 on the occlusal surfaces of the upper permanent molars (17.0%), on the buccal surfaces of the lower permanent molars (16.3%), and on the occlusal surfaces of the lower permanent molars (13.6%). The highest ICDAS codes (4–6) were clearly most common on the second lower primary molars (3.3–5.6%). 

When analyzing the association of the distributions of ICDAS codes on the different surfaces between the first permanent molars and the second primary molars, the strongest correlations were on the lingual surfaces of the maxillary molars and on the buccal surfaces of mandibular molars (Table 3). The correlations were also significant or highly significant on the occlusal surfaces of the maxillary and mandibular molars and on the lingual surfaces of the mandibular molars. 

**Figure 1:**
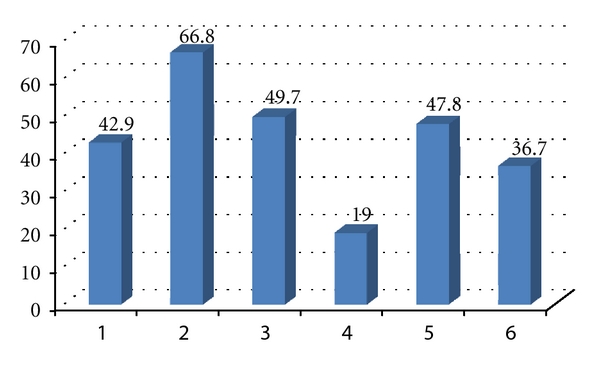
The prevalence (%) of each ICDAS code (>0) per child.

**Figure 2:**
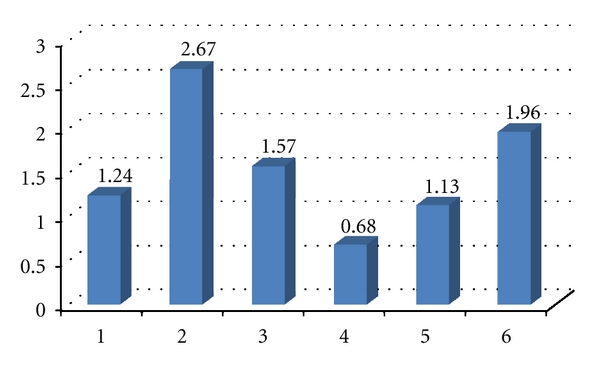
The mean number of surfaces per child with each ICDAS code (>0).

The most prevalent ICDAS codes (>0) per child were the codes 2 and 5 (Figure 1), and the least prevalent code was 4. The highest mean number of surfaces per child with each individual ICDAS codes (>0) was the codes 2 (2.67) and 6 (1.96), and the lowest code was 4 (Figure 2).

All the mean caries indeces was not statistically different in the different age groups or grades (Table 4). However, the mean number of dentine caries lesions (d/D_4–6_T, d/D_4–6_S) seemed to decrease according to increasing age and was lower among the second grade children than among the first graders.

There were also statistically significant differences between the schools (Table 5) in the mean numbers of enamel (d/D_1–3_T, d/D_1–3_S) and dentine caries lesions (d/D_4–6_T, d/D_4–6_S) and restorations (f/FT, f/FS), but not in the mean numbers of caries experience teeth (dmft/DMFT) or surfaces (dmfs/DMFS). 

## 4. Discussion

This study was conducted among the first and second grade children in 10 primary schools of Southeast Estonia, where only a few epidemiological studies have been conducted. All earlier studies from Estonia have used WHO criteria in detecting caries [27–29] and this is the first study in Estonia to use ICDAS method. ICDAS is currently a recommended method globally to assess caries in dental studies [24]. The enamel caries lesions can still be healed by demineralization process, but more prospective studies are needed to determine how many lesions will remineralize and how many from the unhealed lesions do or do not progress. ICDAS is especially valuable method for detecting the enamel caries lesions for planning the individual remineralization therapy or for monitoring the caries pattern at the population level. 

This study confirmed the high caries level in this region. The total caries experience indicators are normally higher in the mixed dentition, because the primary teeth have been exposed longer for the risk factors of dental caries, for example, frequent sugar snacks, drinks, and sweets. This longer exposure time also explains why the primary molars had higher mean number of dentine caries lesions than the permanent molars. The second primary molars have been shown to be more affected than the first primary molars [8]. Therefore, they were compared with the ICDAS codes of the first permanent molars in this study. The higher number of enamel caries lesions in the permanent molars can be explained with the high caries risk period soon after the eruption of the teeth, when the maturation of the enamel takes place. Especially the occlusal surfaces are susceptible and almost one-third of them in this study had already enamel caries. However, the number of these enamel caries lesions was not associated with age or grade of the children. Some of the lesions have obviously already been remineralized. 

High number of caries on the primary molars could be expected to predict caries in the permanent molars. However, in this cross-sectional study, only the associations could be studied. The association of the number of caries lesions between the first permanent molars and the second primary molars was analyzed by Spearman correlation coefficient. Correlation was statistically significant on the occlusal and lingual surfaces on all quadrants and on the lingual surfaces of all quadrants, except between the teeth 16 and 55. The correlation coefficients were not high, but this obviously follows from the lower prevalence of the caries lesions in the first permanent molars, which have not been exposed as long as the second primary molars. The proximal surfaces of the first permanent molars did not had caries lesions at this age. However, for some reason, the correlation was significant on the distal surfaces of the teeth 46 and 75. 

There were no statistical differences in the mean number of dentine caries lesions according to the age. The older children could have been expected to have higher caries experience than the younger ones. However, the older children and the children in the second grade had less dentine caries than the younger ones and the children in the first grade. This could be a warning sign of the rapidly changing diet. Estonia has faced quick changes, when it became independent second time and a member of the European Union in 2004. On average, 3.9–4.3 teeth/child had been restored in all age groups and 5.7–6.0 teeth/child had caries experience. These can be considered very high caries indicators. Similarly, high caries levels have been reported from all the Baltic countries, Estonia [27, 28], Latvia [30], and Lithuania [30, 31]. 

All the caries indicators (except caries experience) seemed to differ significantly between the schools. One school, Elva, had the highest mean caries experience and highest mean number of untreated obvious carious teeth and surfaces. However, the differences in caries experience were not statistically different, which might be explained by the equal availability of the restorative treatment. The high number of restorations in two schools (Melliste and Rõngu) might reflect overtreatment. 

The distribution of the ICDAS codes is difficult to explain. The code 2 (enamel caries detected on a wet tooth surface) might be easier to detect than the code 1 (enamel caries visible only when tooth surface is dry) and code 3 (minor cavitation on enamel only). This has been confirmed also in the other ICDAS studies [21]. It has been shown earlier that the ICDAS method requires more time for assessment than the WHO method [19], but the difference is quite small because by both methods every surface needs to be assessed. 

It can be concluded that the ICDAS method gives much more relevant information about caries process than WHO method, when the enamel caries lesions can consistently be detected. The distribution of the ICDAS codes correlated between the primary and permanent molars of the mixed dentition.

## Figures and Tables

**Table 1:** Distribution of the children by age, grade, and gender.

	*n*	Girls (%)	Boys (%)
Age			
7	166	35.3	33.3
8	253	51.1	53.0
9	66	13.6	13.7
Total	485	100.0	100.0

Grade			
I	224	48.4	44.3
II	261	51.6	55.7
Total	485	100.0	100.0

**Table 2:** The mean percentages of ICDAS codes in the upper and lower first permanent molars and second primary molars according to tooth surfaces.

	ICDAS codes						
	0	1	2	3	4	5	6
16/26							
M	97.7	0.3	0.4	0.3	0.0	0.2	0.0
O	62.5	12.4	17.0	4.8	0.8	1.4	0.0
D	99.0	0.0	0.0	0.0	0.0	0.0	0.0
B	91.5	2.2	4.8	0.3	0.0	0.1	0.0
L	83.4	4.7	9.0	1.0	0.3	0.5	0.0

55/65							
M	81.8	0.4	1.5	1.0	1.4	4.3	5.4
O	69.4	5.1	10.2	3.5	1.0	2.9	2.3
D	93.4	0.1	0.1	0.7	0.3	1.2	4.0
B	90.9	0.6	1.4	0.3	0.1	0.4	2.1
L	83.9	2.2	3.1	2.1	0.5	0.8	3.3

36/46							
M	94.6	0.3	3.6	0.4	0.0	0.2	0.2
O	68.8	7.5	13.6	6.4	1.1	1.5	0.3
D	99.1	0.0	0.0	0.1	0.0	0.0	0.1
B	64.5	9.4	16.3	7.3	0.6	0.9	0.2
L	97.3	0.5	1.3	0.0	0.0	0.0	0.1

75/85							
M	72.9	0.9	5.0	3.0	0.5	3.7	5.6
O	66.6	4.1	8.9	3.5	1.0	2.2	5.3
D	82.9	0.3	1.0	0.0	0.0	1.2	6.0
B	68.0	3.3	13.1	2.0	0.1	0.5	3.5
L	85.0	0.2	0.8	0.1	0.1	0.4	4.9

**Table 3:** The Spearman correlation coefficients (*r*) between the first permanent molars and the second primary molars according to the surfaces.

Teeth no.	Surfaces	*r*	*P* value	Teeth no.	*r*	*P* value
16/55	M	0.00	0.936	26/65	−0.04	0.414
	O	0.15	0.001		0.13	0.006
	D	0.00	1.000		0.00	1.000
	B	0.08	0.076		0.09	0.040
	L	0.19	<0.0001		0.14	0.003

36/75	M	0.03	0.540	46/75	0.16	0.001
	O	0.19	<0.0001		0.23	<0.0001
	D	0.00	1.000		0.15	0.002
	B	0.25	<0.0001		0.22	<0.0001
	L	0.14	0.003		0.16	0.001

**Table 4:** The caries indices by age and grade.

	d/D_1–3_T	d/D_1–3_S	d/D_4–6_T	d/D_4–6_S	f/FT	f/FS	dmft/DMFT	Dmfs/DMFS
Age								
7	3.7	4.6	2.2	4.0	3.9	6.4	5.9	12.0
8	3.8	5.1	1.8	3.3	4.2	6.7	6.0	11.9
9	4.3	5.7	1.5	2.2	4.3	7.2	5.7	11.4
Mean	3.8	5.0	1.9	3.4	4.1	6.7	5.9	11.9
P*	0.411	0.339	0.281	0.220	0.785	0.657	0.653	0.685

Grade								
I	3.7	4.7	2.3	4.1	3.9	6.3	6.0	12.3
II	3.9	5.3	1.6	2.7	4.3	7.0	5.9	11.5
Mean	3.8	5.0	1.9	3.4	4.1	6.7	5.9	11.9
P*	0.565	0.374	0.053	0.101	0.150	0.112	0.403	0.304

*Kruskal-Wallis test.

**Table 5:** The caries indices according to the schools.

School	d/D_1–3_T	d/D_1–3_S	d/D_4–6_T	d/D_4–6_S	f/FT	f/FS	d/D_4–6_MFT	d/D_4–6_MFS
Lähte	2.2	2.7	2.1	4.0	3.9	5.6	5.6	10.3
Tõrva	4.0	5.0	1.6	2.1	3.6	6.3	5.2	10.6
Melliste	3.5	4.4	1.4	2.1	5.0	8.3	6.1	11.9
Räpina	4.3	5.6	2.7	5.2	4.0	6.4	6.4	12.7
Võnnu	3.9	5.1	2.9	4.6	3.7	5.8	6.3	11.9
Rõngu	4.0	5.1	1.5	3.4	4.9	7.9	6.4	12.8
Võru	4.0	5.4	0.9	1.5	4.4	7.7	5.5	11.6
Elva	4.0	5.4	3.4	7.5	2.9	4.6	6.5	14.9
Nõo	4.4	5.9	2.1	4.0	3.9	5.7	6.2	12.9
Descartes	3.8	5.5	1.8	3.2	2.8	4.4	4.8	9.6
P*	0.018	0.011	<0.001	<0.001	0.011	0.006	0.302	0.601

*Mann-Whitney *U* test.
